# Investigation of the Thermophysical Simulation and Material Removal Mechanism of the High-Volume-Fraction SiCp/Al Composite in Wire Electrical Discharge Machining

**DOI:** 10.3390/ma17225546

**Published:** 2024-11-13

**Authors:** Zhi Chen, Jiawen Hu, Hongbing Zhou, Yumeng Wei, Guojun Zhang, Fenglin Han

**Affiliations:** 1 State Key Laboratory of Precision Manufacturing for Extreme Service Performance, College of Mechanical and Electrical Engineering, Central South University, Changsha 410083, China; 217052@csu.edu.cn (Z.C.); 223711005@csu.edu.cn (J.H.); zhouhongbing196@gmail.com (H.Z.); 193712196@csu.edu.cn (Y.W.); 2 Guangdong Provincial Key Laboratory of Manufacturing Equipment Digitization, Guangdong HUST Industrial Technology Research Institute, Dongguan 523808, China; zhanggj@hust.edu.cn

**Keywords:** WEDM, SiCp/Al composite, material removal mechanism, surface roughness prediction, high volume fraction

## Abstract

SiC particle reinforced aluminum matrix composites (SiCp/Al) are widely used in aviation, weaponry, and automobiles because of their excellent service performance. Wire electrical discharge machining (WEDM) regardless of workpiece hardness has become an alternative method for processing SiCp/Al composites. In this paper, the temperature distribution and the discharge crater size of the SiCp/Al composite are simulated by a thermophysical model during a single-pulse discharge process (SPDP) based on the random distribution of SiC particles. The material removal mechanism of the SiCp/Al composite during the multi-pulse discharge process (MPDP) is revealed, and the surface roughness (*Ra*) of the SiCp/Al composite is predicted during the MPDP. The thermophysical model simulation results during the MPDP and experimental characterization data indicate that the removal mechanism of SiCp/Al composite material consists of the melting and vaporization of the aluminum matrix, as well as the heat decomposition and shedding of silicon carbide particles. Pulse-on time (*T_on_*), pulse-off time (*T_off_*), and servo voltage (*SV*) have a great influence on surface roughness. The Ra increases with an increase in *T_on_* and *SV*, but decreases slightly with an increase in *T_off_*. Moreover, compared with experimental data, the relative error of *Ra* calculated from the thermophysical model is 0.47–7.54%. This means that the developed thermophysical model has a good application and promotion value for the WEDM of metal matrix composite material.

## 1. Introduction

SiCp/Al is a composite material composed of aluminum (Al) matrix and silicon carbide (SiC) particles. Aluminum matrix has excellent specific strength, thermal conductivity, and plasticity. Silicon carbide particles have ultra-high hardness and wear resistance [1,2,3]. Therefore, SiCp/Al composite materials not only have excellent mechanical properties, but also have good thermal conductivity and wear resistance, suitable for various high-load, high-temperature, and corrosive environments [4,5]. However, due to the high wear resistance and hardness of SiCp/Al, traditional cutting methods applied to SiCp/Al composites are prone to problems such as low machining efficiency, severe tool wear, and poor machining accuracy and surface quality. In recent years, diamond tools have effectively processed SiCp/Al composites [6,7]. However, the high processing cost of diamond cutting tools severely limits the application of SiCp/Al composite materials, especially for SiCp/Al composites with a high SiC content.

EDM/WEDM is an electrical removal machining process that can process conductive materials of any hardness with low residual stress [8,9]. Therefore, EDM/WEDM can be considered an effective processing method for high-hardness and wear-resistant SiCp/Al composite materials [10]. In the past twenty years, many researchers have attempted to use EDM/WEDM to process SiCp/Al with various SiC particle contents. Kumar S.S. et al. explored the effects of discharge machining parameters on the machining characteristics of Al-SiC (5 wt.%)-B_4_C in EDM. Their experimental data indicated that discharge current was a key factor of electrode wear, *Ra*, and power consumption [11]. Wang Z.L. et al. used micro WEDM to process 35–45 vol.% SiCp/Al composite materials. Through multiple trim cutting, an *Ra* of 0.7 μm and a recast layer of 3 μm were obtained [12]. Naik S. and Yadav R.N. et al. investigated the relationship between discharge factors with a machining efficiency and *Ra* of 10–16% SiCp/Al in WEDM. Numerical models of processing parameters and process indicators were established to search for the best discharge factors [13,14]. Dhar S. et al. proposed low-temperature EDM to process 10 wt.% SiCp/Al composites. Experimental data showed that low-temperature devices could effectively increase the processing efficiency and reduce electrode wear [15]. Mohan B. added rotating tube electrodes in the EDM of 20–25 wt.% SiCp/Al. Their experimental results showed that, compared to ordinary electrodes, rotating tube electrodes could effectively improve the expelling of discharge erosion residue, thereby improving machining efficiency and reducing the electrode wear rate [16]. The above research has proved that EDM/WEDM is a feasible processing method for SiCp/Al. Optimizing machining parameters or improving machining processes (such as low-temperature assistance and rotating tube electrodes) [17] could effectively improve machining performance. However, most of the research objects in the above studies were SiCp/Al composites with low SiC content. SiCp/Al composite materials with high SiC content have lower conductivity, which results in a poorer discharge state and makes processing more difficult.

Many researchers have made great efforts and progress in developing novel, efficient, and accurate phase-field models [18]. Singh et al. [19] used an auxiliary electrode for the EDM of non-conductive alumina and established a 2D axisymmetric FEM to obtain temperature field distribution. Jithin et al. [20] improved the stochastic continuous pulse model of EDM by establishing multiple Gaussian heat sources with random positions, energies, and times. Compared with the even and general thermal distribution model, the prediction accuracy of the random multi-heat source model was improved, with an average prediction error of about 11.5%. Shahane et al. [21] improved the simulation of random continuous pulse discharge. Taking into account the overlap effect between current and previous discharge sparks, a simulation model for WEDM was established for predicting the size of discharge craters. If the current discharge point was in the same position as the previous discharge point, the discharge crater would become smaller. Their experimental data indicated that the simulation model had high prediction accuracy (a relative error of 2.7–9.4%). Burlayenko et al. [22] derived the plane strain finite element expression of functionally graded materials through a finite element integration matrix. This model and equation had high reliability in predicting temperature field and thermal stress distribution. Tang et al. [23] established a single-pulse thermo-electric coupling model for powder-mixed EDM. According to the simulation results in SPDP, a thermo-electric coupling model for continuous pulse discharge machining was established using a random heat source distribution model. This model was proved to have high predictive accuracy for the processing efficiency of 5 wt.% SiCp/Al composite materials in PMEDM. Ming et al. [24] built a thermophysical etching model in WEDM of BN-AIN-TiB2 composite ceramic materials with different mass fractions. The built model could be used to predict the material removal rate, *Ra*, and slit width, and could determine the optimal processing parameters based on the predicted results. The above simulation models for the EDM of multiphase materials were mainly used to predict temperature field distribution, machining efficiency, and *Ra*. There is relatively little research on the removal mechanism of multiphase materials in EDM. In addition, in most models, multiphase materials were simplified as single-phase materials or uniformly distributed ceramic particles. The accuracy of these simulation models is relatively low. 

WEDM has been proved to be an alternative method for processing SiCp/Al. However, the random distribution of insulating and superhard SiC particles makes the processing of SiCp/Al difficult [25]. Therefore, it is difficult to reveal the material removal mechanism and to forecast the surface roughness (*Ra*), especially for SiCp/Al materials with high SiC content. Considering the random distribution of SiC particles, it is necessary to further establish a more accurate simulation model. Aiming at these difficulties, a multiphase particle random distribution thermophysical model is established, and the simulation of single-pulse and multi-pulse discharge is carried out in this paper. This model can directly and clearly reflect the removal mechanism of SiCp/Al composite particle composites in WEDM, and the reliability of this model in predicting discharge pits and Ra is proved by experimental comparison.

## 2. Experimental and Test Section

### 2.1. Material

SiCp/Al with different SiC contents has different material properties, which can adapt to different usage requirements. When WEDM is used to process SiCp/Al, the material conductivity and the flow of discharge debris decrease with the increase in SiC content. At the same time, this also reduces the proportion of effective discharge states, which can lead to more complex machining processes and reduce machining efficiency and surface quality. The revelation of the material removal mechanism and the prediction of surface roughness will become more difficult.

**Figure 1:**
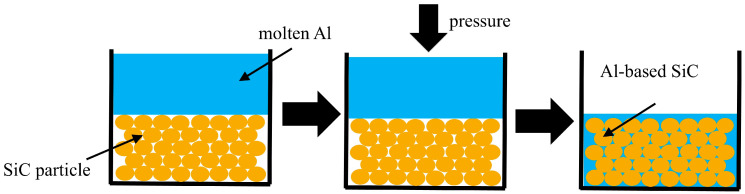
The preparation principle of SiCp/Al composites by pressure casting.

The object of this study was SiCp/Al with an SiC content of 65%. In this study, the method of pressure casting was chosen as the preparation method for SiCp/Al [26,27]. The preparation principle is represented in Figure 1: (a) SiC particles are placed in the forming cavity. (b) The molten aluminum substrate is sprayed into the cavity through a high-speed and high-pressure nozzle. (c) A certain pressure is applied to the molten aluminum substrate and stability is maintained for a period of time. During this process, the molten aluminum matrix fills the gaps of SiC particles. (d) After cooling, multiphase composite materials can be obtained. The thickness of each test sample is 4 mm.

### 2.2. Machine Tool

**Figure 2:**
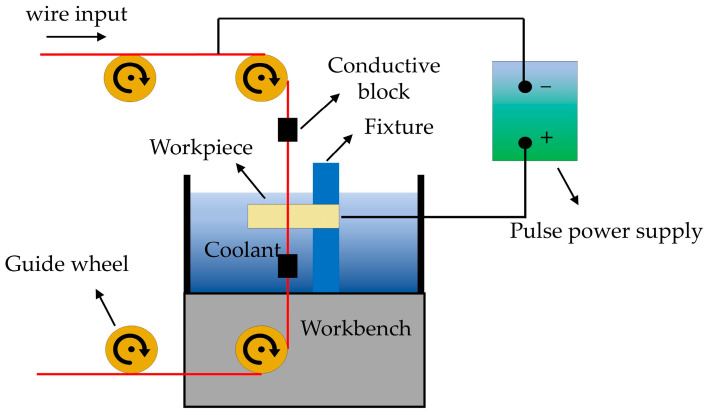
The schematic drawing of machining equipment.

All WEDM experiments were completed on a five-axis low-speed WEDM machine tool (ACCUTEX Au-300i, Taiwan, China). The schematic diagram of the machine is shown in Figure 2, which mainly includes a CNC system, the discharge system, the wiring system, and the workbench. Table 1 lists the main technical parameters of machining equipment. During discharge processing, the workpiece was immersed in deionized water, and the electrode wire was a 0.25 mm diameter brass wire.

In this section, the relatively appropriate process parameters are selected based on previous studies [28]. With pulse-on time (*Ton*), pulse-off time (*Toff*), servo voltage (*SV*), wire-speed (*WS*), and wire tension (*WT*) as parameters, the cutting experiment was completed to study the machining properties of 65 vol.% SiCp/Al composites. By measuring the *MRR* and *Ra*, characterizing the material surface and analyzing the chemical composition, the appropriate discharge factors were found. Other constant process parameters were as follows: discharge average current 15 A, discharge peak voltage 80 V, maximum forward speed 16 mm^2^/min, and cutting length 10 mm.

### 2.3. Test Method

The calculation formula of the material removal rate (*MRR*) is shown in Equation (1) [29]. *L* is the cutting length, which is fixed at 10 mm in this study. *H* is the workpiece thickness, which is fixed at 4 mm in this study. *t* is the processing time (unit: s).

(1) $$\mathrm{MRR}=\frac{LH}{t}\cdot \;(m^{2}/s)$$

The models and parameters of experimental measuring equipment are shown in Table 2. An optical profilometer (WYKO NT9100, New York, NY, USA) was used to detect the *Ra* of the processed surface. The *Ra* on the sample surface of each sample was measured three times. The average of three measurements was taken as the final measurement value. A field-emission scanning electron microscope (SEM, MIRA 3 LMU, Brno, Czech Republic) was used to detect the microstructure and elemental composition on the sample surface. The observation multiple of the microtopography of the processed surface was 1000×. The chemical composition was analyzed by SEM with energy dispersive spectroscopy (EDS). This determines the chemical composition of a sample by analyzing the X-rays that different elements emit at specific energies when subjected to an electron-beam laser. An X-ray diffractometer (XRD, Bruce D8 ADVANCE, Saarbrucken, Germany) was used to detect the phase composition on the sample surface using Cu Kα radiation within a 2θ range from 20 to 80°. The scanning step size was 0.045° per step. 

## 3. The Development and Analysis of the Thermophysical Model During SPDP

### 3.1. Modeling During SPDP

#### 3.1.1. Establishment of Multiphase Material Model

**Figure 3:**
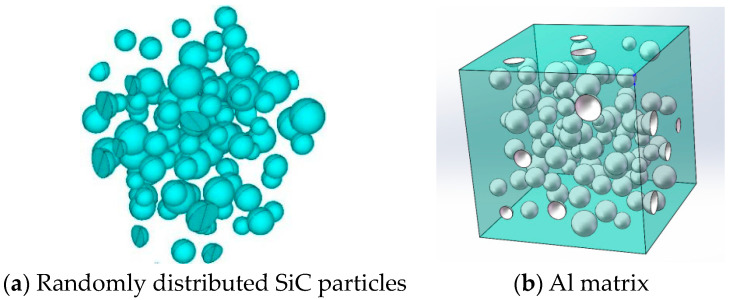
The multiphase material model of SiCp/Al.

In practical applications, the size of SiC particles in SiCp/Al composites is inconsistent. SiC particles are randomly distributed in the Al matrix. Therefore, to develop a more realistic model of SiCp/Al, as shown in Figure 3, a finite element mode of the randomly distributed SiC particles was automatically generated by combining C language and ANSYS APDL programming. The modeling process is as follows:

1. All SiC particles are assumed to be regular spheres with random radii and random position distributions. To accelerate the calculation speed, the Al matrix is a regular cube with a side length of 1 mm. The radius range of SiC particles is 5 to 50 μm.
2. SiC particles cannot exceed the boundary surface of the Al substrate. If an SiC particle is located at the boundary of the Al matrix, this SiC particle will be cut open. Only the portion of the aluminum matrix is retained.
3. Boolean operations in ANSYS 2018 are adopted to prevent SiC particles from overlapping with the Al matrix.

#### 3.1.2. Key Factors of Thermophysical Model

1. Model Assumptions

SiC particles and Al matrix were set as surface contact pairs. The contact unit of SiC particles is CONTACT174 on the target surface, and Al is CONTAT173 on the target surface.

In order to shorten the simulation calculation time, the simulation model was simplified as follows [30]:

(a) The thermal energy converted from discharge energy follows the Gaussian distribution. Thermal conduction and thermal convection are considered the method of thermal transmission.
(b) The physical properties of the workpiece material are only related to temperature. Ignore the thermal expansion effect. The shape of the workpiece remains unchanged.
(c) After the pulse discharge is completed, the discharge channel restores its insulation state under the action of deionized water.

2. Heat source

Based on Fourier’s law, the amount of thermal conduction per unit time through a unit cross-sectional area (the heat flux *q*) is proportional to the rate of temperature change perpendicular to that cross-section, as shown in Equation (2) [31]:

(2) $$q=-\lambda A\frac{dT}{d\delta }\cdot (W/m^{2})$$

*dT/dδ* is the temperature gradient (unit: K/m). *A* is the thermal conduction area. *q* is the thermal conduction of an object per unit time. *λ* is the thermal conductivity. The direction of thermal transfer is opposite to the temperature gradient.

In WEDM, due to the movement of the electrode, there is relative movement between the dielectric and the workpiece surface. Then, a part of the discharge thermal energy will be transmitted to the dielectric through thermal convection, as shown in Equation (3) [31]:

(3) $$q=h(t_{w}-t_{f})=h\Delta t\cdot (W/m^{2})$$

According to the discharge mechanism of EDM, the discharge energy is considered by most researchers as a Gaussian distribution heat source. The mathematical expression of the Gaussian heat source is shown in Equation (4) [26]:

(4) $$q(r)=\frac{3f_{c}U_{\left(t\right)}I_{\left(t\right)}}{\pi R^{2}}\exp\left\{-3{\left(\frac{r}{R_{p}}\right)}^{2}\right\}$$

*f_c_* is the energy absorption coefficient of the workpiece, as shown in Equation (5) [32]. *U_(t)_* is the discharge voltage, *I_(t)_* is the discharge current, and *R* is the discharge radius.

(5) $$f(c)=5.672+0.2713\times I^{0.5598}T_{on}^{0.4602}$$

The discharge radius depends on *T_on_* and the discharge current, as shown in Equation (6) [33]:

(6) $$R_{p}=2.04\times 10^{-3}I_{\left(t\right)}^{0.43}T_{on}^{0.44}$$

3. Boundary condition

**Figure 4:**
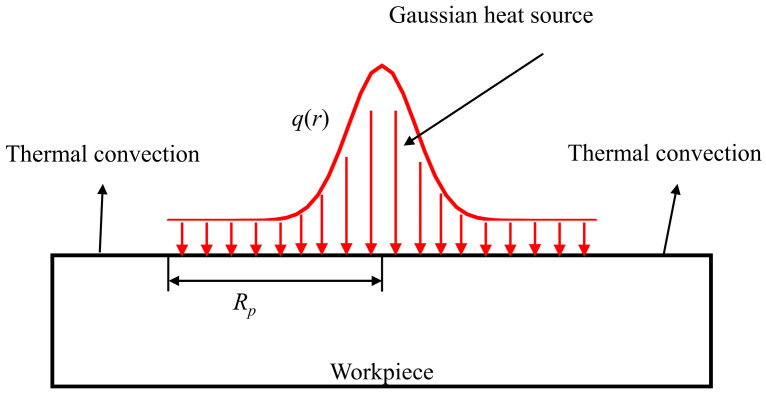
The boundary conditions of the thermophysical model.

The boundary conditions of EDM are shown in Figure 4. Due to the combined effects of heat conduction and convection, the thermal boundary function is a stepped function related to the radius, as shown in Equation (7) [34]. When the radius is smaller than the discharge radius, both thermal conduction and thermal convection exist simultaneously. When the radius is greater than the discharge radius, only thermal convection exists.

(7) $$\lambda \frac{\partial T}{\partial r}=\left\{\begin{array}{l} -h\left(T-T_{0}\right),r>R_{p}\\ q\left(r\right)-h\left(T-T_{0}\right),r\leq R_{p} \end{array}\right.$$

where *λ* is thermal conductivity. *T* is the temperature. *T*_0_ is room temperature. *h* is the coefficient of thermal convection. *R* is the discharge radius.

4. Material thermal properties

The material physical parameters are represented in Table 3 and Table 4.

### 3.2. Simulation and Analysis

**Figure 5:**
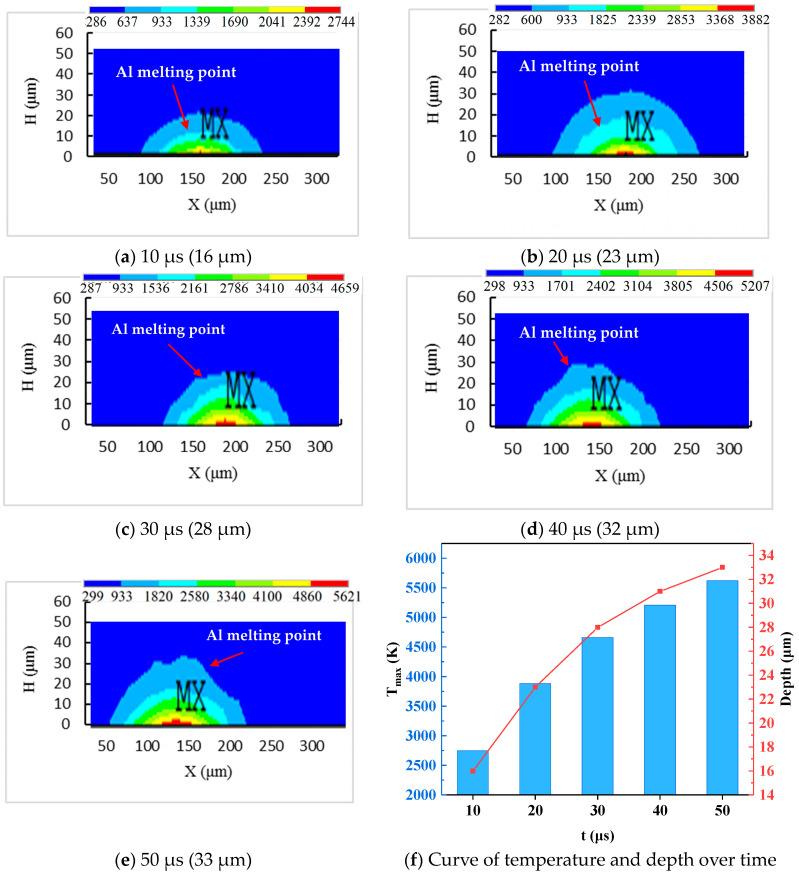
The temperature distribution in the depth direction at different times (“MX”: the highest temperature region in the temperature field).

The process of EDM includes discharge, material removal, and detrital flow. When the gap between the electrode and the workpiece reaches a certain level, the working medium in the gap is ionized under the action of the electric field, and a plasma channel is formed. Then, the conductivity of the gap is greatly enhanced. Electric spark discharge forms inside the plasma channel. After that, a violent collision occurs between electrons, ions, and the workpiece surface in the high-temperature plasma channel. Intense collisions generate a large amount of heat, causing a rapid increase in temperature within the plasma channel. Figure 5 exhibits the temperature distribution in the depth direction of the workpiece at different times. The process factors include a *T_on_* of 50 μs, a discharge peak current of 15 A, and an *SV* of 45 V. It can be observed that at the 10 μs moment, the highest temperature of the workpiece is 2744 K, and the melting depth of the aluminum substrate is 16 μm. As the discharge time increases, the maximum temperature and the melting depth of the aluminum substrate also increase. At the 50 μs moment, the maximum temperature is 5621 K, and the melting depth of the aluminum substrate is 33 μm. This is because of the fact that the maximum temperature of the workpiece is 5621 K, which exceeds the melting and boiling temperatures of the Al matrix and also exceeds the heat decomposition temperature of silicon carbide. The removal method of SiCp/Al composite material should include the melting or vaporization of the Al matrix and the heat decomposition of SiC particles.

**Figure 6:**
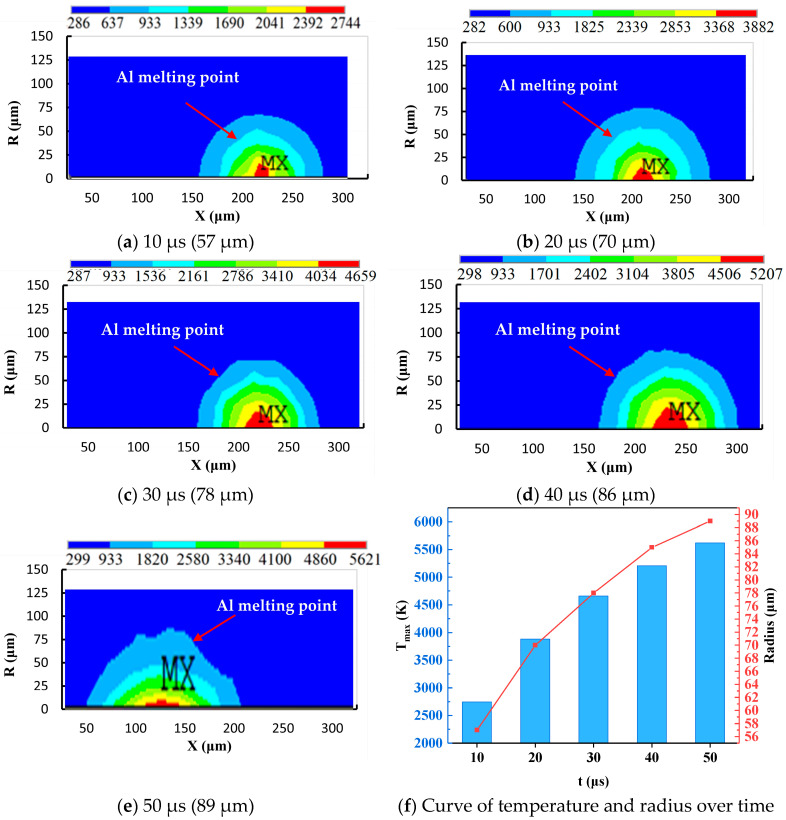
The temperature distribution along the radius direction at different times (“MX”: the highest temperature region in the temperature field).

Figure 6 shows the temperature distribution along the radius direction of the workpiece at different times. The process factors include a *T_on_* of 50 μs, a discharge peak current of 15 A, and an *SV* of 45 V. It can be observed that at the 10 μs moment, the melting radius of the aluminum substrate is 57 μm. As the discharge time increases, the melting radius of the aluminum substrate also increases. At the 50 μs moment, the melting depth of the aluminum substrate is 89 μm. Combining Figure 5 and Figure 6, the entire discharge crater can be approximated as an ellipsoid. The radius direction and depth direction are the major and minor axes of the ellipse, respectively. In addition, as the discharge time increases, the volume of the ellipse first increases rapidly and then increases slowly.

**Figure 7:**
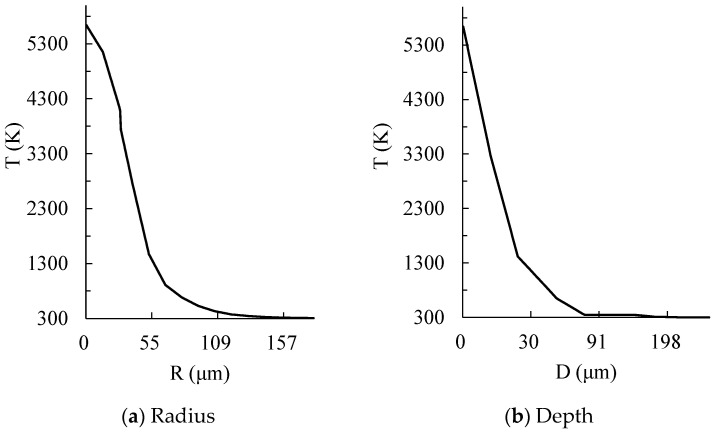
The temperature curve in the radius and depth direction (*T_on_*: 50 μs).

Figure 7 shows the temperature curve in the radius and depth directions. The discharge parameters include a *T_on_* of 50 μs, a discharge peak current of 15 A, and an *SV* of 45 V. From Figure 7, it can be seen that the material temperature drops with the increase in discharge radius and depth. The highest temperature on the machined surface is obtained at the discharge center point. Figure 7a represents the temperature distribution of the workpiece in the radial direction. It can be observed that, when the radius is less than 12 μm, the temperature of the workpiece decreases relatively slowly. When the radius is greater than 12 μm, the temperature of the workpiece decreases rapidly. This phenomenon is mainly due to the fact that, within the discharge radius, the heat flux variation in the workpiece includes the input of discharge energy and the output of thermal convection. Beyond the discharge radius, the heat flux variation in the workpiece only includes the output of thermal convection. Figure 7b shows the temperature distribution in the depth direction. It can be observed that when the depth is less than 26 μm, the workpiece temperature rapidly decreases along the depth direction. When the depth is greater than 26 μm, the temperature of the workpiece slowly decreases until room temperature. This phenomenon is mainly because the heat transfer mode in the depth direction only includes heat conduction, and the thermal transfer is directly related to temperature gradient. At a depth of 26 μm, due to the influence of phase change, the rate of temperature change undergoes a sudden change.

## 4. The Development and Analysis of the Thermophysical Model During MPDP

### 4.1. Modeling During MPDP

According to the thermophysical model during the SPDP, the size of the discharge crater in the SPDP is in the micrometer range. In actual WEDM, hundreds to thousands of pulses are applied to the workpiece surface every second. In addition, this is due to the fact that electrode and workpiece surfaces cannot be absolutely smooth. Therefore, the principle of continuous pulse discharge machining is similar to that of SPDP machining. Due to the large and unpredictable number of discharge points, the material removal process of continuous pulse discharge is more complex.

In order to shorten the simulation calculation time, the simulation model was simplified for continuous pulse discharge as follows [36]:

(a) The heat source model and boundary conditions of the MPDP are consistent with those of the SPDP.
(b) Only one plasma channel is formed within each pulse discharge cycle. The position of each plasma channel is randomly distributed. The discharge channels of multiple pulses do not affect each other.
(c) The physical properties of the workpiece material are only related to temperature. Ignore the thermal expansion effect. The shape of the workpiece remains unchanged.
(d) After the pulse discharge is completed, the discharge channel restores its insulation state under the action of deionized water.

### 4.2. Simulation and Analysis

The thermophysical model simulation steps for the MPDP of SiCp/Al composite material are as follows: Step 1: Through C language, a randomly distributed Gaussian heat source is generated. Step 2: This thermal source is loaded onto the machined surface to obtain a discharge crater. Step 3: Based on the melting point of the Al matrix and heat decomposition temperature of SiC, the “life and death unit method” is used to kill the grids of aluminum materials and SiC, which are melted or thermally decomposed. Thus, the thermal simulation during the one-pulse discharge process is completed. Step 4: One should wait for a pulse off time and repeat Step 1. 

**Figure 8:**
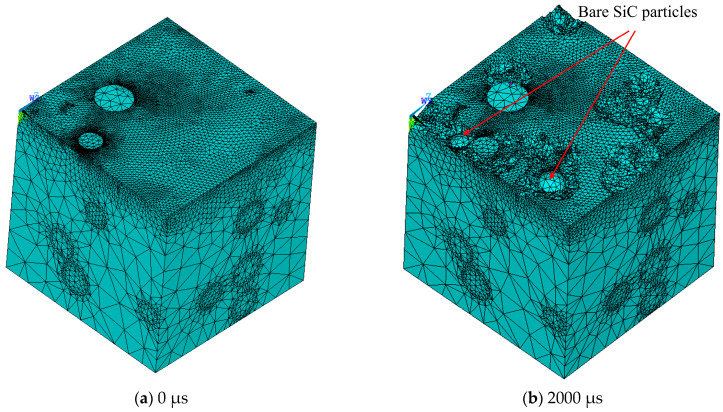

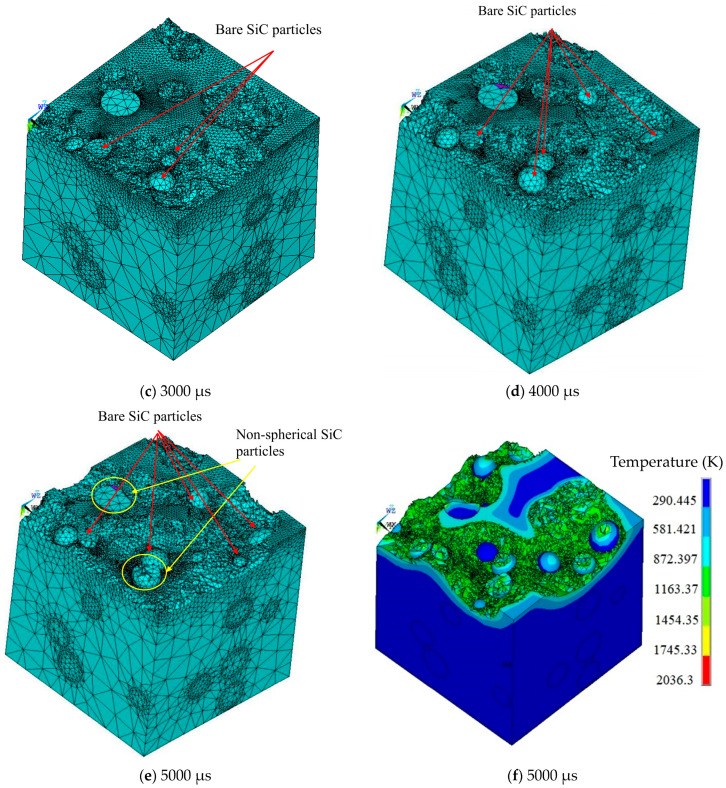
The discharge microtopography after MPDP machining at different times.

Figure 8 shows the discharge microtopography after MPDP machining at different times. The discharge factors are as follows: *T_on_* = 50 μs, *I* = 10 A, *U* = 45 V, and *T_off_* = 30 μs. In Figure 8a, only two SiC particles are located on the simulation unit surface. In Figure 8b, some of the aluminum matrix disappears on the surface of the simulation unit, and two SiC particles are exposed due to the melting or vaporization of the aluminum matrix. As the discharge time increases, more and more Al substrates disappear. At the same time, more and more SiC particles with increasing volumes are exposed. In Figure 8e,f, some non-spherical SiC particles are found. These phenomena are mainly due to the fact that, according to the thermophysical properties of Al and SiC, the heat decomposition temperature of SiC is much higher than the melting point of Al, and even higher than the boiling point of Al. So, when the discharge energy is loaded on the workpiece surface, the Al matrix will first be melted or vaporized and absorb a large amount of heat. When the temperature exceeds the heat decomposition temperature of SiC, some SiC particles will be thermally decomposed. When most of the Al matrix around the SiC particles is melted, these SiC particles will fall off into the dielectric under the action of gravity or dielectric erosion.

## 5. The Characterization of the Machined Workpiece Surface

**Figure 9:**
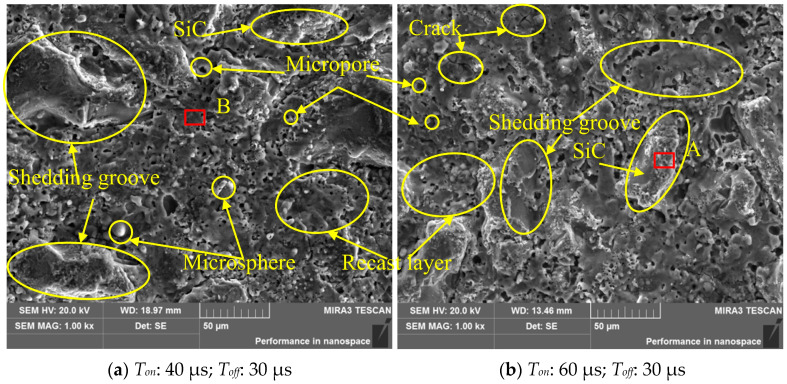
SEM results on machined surface.

Figure 9 shows the SEM results of the sample surface. From Figure 9, it can be seen that cracks, microspheres, micropores, and recast layers appeared on the machined surface. The principle of WEDM is similar to that of EDM. When a large amount of concentrated discharge heat acts on the workpiece surface, the workpiece material is melted or vaporized under the high temperature. The melted or vaporized workpiece material is thrown out because of the action of impact, electric field, and flow field. Then, a discharge crater is generated on the machined surface. Under the cooling effect of the medium, the thrown workpiece material is transformed back into solid debris. Most of the discharge debris is expelled by the deionized water flushing. A small part of the discharge debris reattaches to the workpiece surface, which results in a complex morphology on the machined surface. These reattached debris become recast layers [37,38]. In addition, due to the randomness of the position of spark discharge points, the uneven insulation medium flow and the large temperature gradient, a certain amount of thermal stress is generated on the machined surface. If the thermal stress exceeds the tensile strength of the workpiece material, microcracks will be formed on the workpiece surface after the cooling effect of dielectric [39,40]. Moreover, a large number of micropores appear on the processed surface, which may come from the remaining gas in the pressure casting process, the bubbles generated during the discharge process, or the plastic deformation caused by the increase or decrease in temperature [41]. Furthermore, some relatively large protrusions (length of about 50 μm) are also found on the workpiece surface, which are larger than those observed in conventional discharge microtopography. These protrusions should be SiC particles. Some smooth and large pits (length of about 50 μm) are also found on the workpiece surface, which are larger than those in the conventional discharge microtopography. These pits should be the shedding grooves of SiC particles.

**Figure 10:**
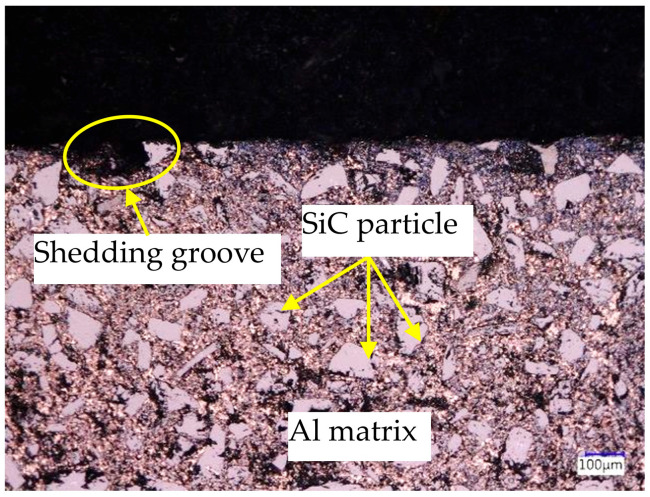
The microscope results on the cross-section.

Figure 10 shows the microscope results of the cross-section of the processed workpiece. From Figure 10, (1) SiC particles are almost uniformly distributed within the aluminum matrix, and the geometric shapes and sizes of SiC particles are various. The geometric size range is 5–50 μm. (2) Some large and deep grooves are observed on the processed surface. The geometric shapes and sizes of these grooves are various. According to the mechanism of EDM, these grooves are shedding grooves of SiC particles rather than discharge craters.

Table 5 shows the EDS results of elemental composition. The results show that (a) in region A of Figure 9, the composition of silicon is much higher than other elements such as Al and Cu. The main component of region A is the exposed SiC particles, and some of the surrounding aluminum matrix has been eroded. (b) In region B, the composition of aluminum and silicon elements is much higher than other elements. It can be inferred that a large amount of aluminum matrix is surrounded by SiC particles in region B. (c) A small amount of O and Cu elements are detected on the processed workpiece surface. The main source of Cu element is the transfer of copper element on the wire electrode. Due to the effect of the high temperature, the wire electrode will also undergo a certain degree of melting due to the high temperature and impact. A small portion of melted Cu will adhere to the machined surface. Al element is prone to undergo oxidation reactions with O element and to form oxides. In areas with high aluminum content, the content of oxygen is also relatively high. (d) The composition ratio of carbon and silicon elements is significantly different from 1:1. This is mainly because SiC particles undergo heat decomposition under high temperatures, forming single-crystal Si and C. Single-crystal SiC is more easily vaporized than C [42].

**Figure 11:**
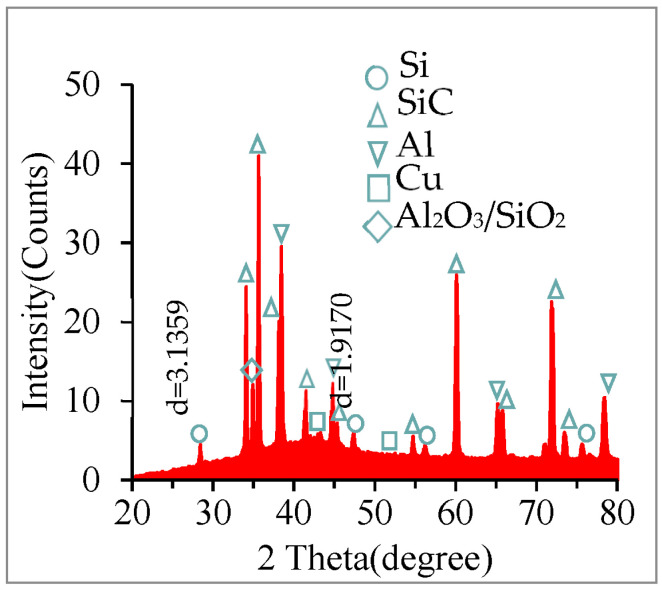
XRD results of chemical compounds.

Figure 11 represents the XRD results of chemical compounds. In XRD detection, the scanning angles of Si peaks are 28°, 47°, and 56°, respectively. The chemical compounds on the machined surface include Si, SiC, Al, Cu, and Al_2_O_3_/SiO_2_. Si crystals come from the heat decomposition of SiC particles because of the discharge energy. In addition, the Al and Si elements on the workpiece surface undergo oxidation reactions with oxygen in air or water [43,44,45,46]. This corresponds to the results shown in Table 5.

## 6. The Prediction of *Ra*

**Figure 12:**
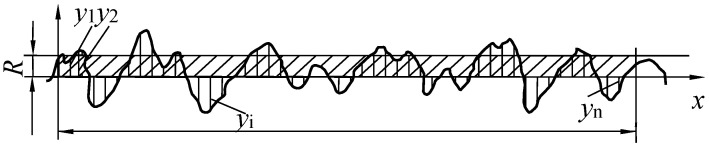
The schematic concept of *Ra*.

**Figure 13:**
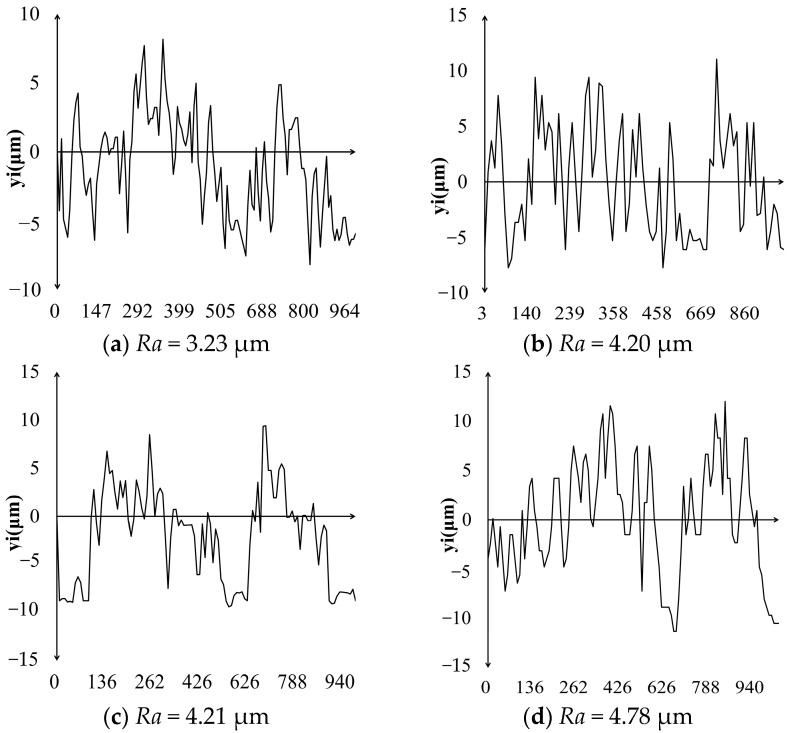
The *Ra* from the thermophysical model during the MPDP.

**Figure 14:**
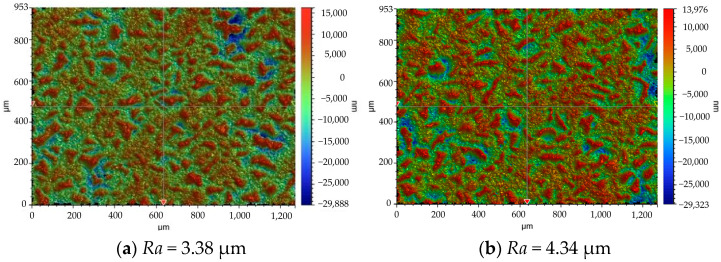

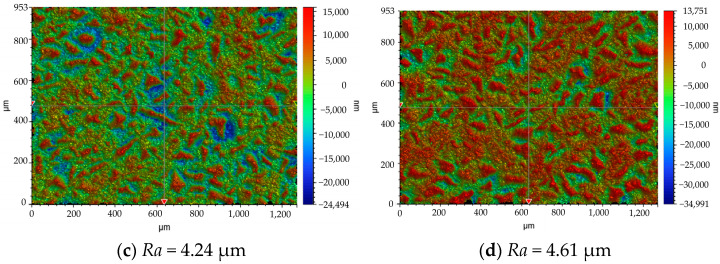
The *Ra* from the experimental measurement data.

Based on the thermophysical model during the MPDP, the three-dimensional digital contour of the discharge microtopography can be obtained, which can be used to obtain the geometric profile of an optional cross-section. Through the least squares method, the median line of the cross-sectional profile can be calculated. The calculation formula for *Ra* (Equation (8)) is the arithmetic average of the absolute value of the longitudinal coordinates from each point on the outline to the center line within a sampling length, and the schematic concept of *Ra* is shown in Figure 12. The *Ra* of this section can be calculated as shown in Figure 13. The experimental measurement result of *Ra* and the corresponding processing parameters are shown in Figure 14. The processing parameters for the experiment are as follows: No. 1–5 set *T_off_*: 30 μs and SV: 45 V, and change *T_on_* to 40, 50, 60, 70, and 80 μs, respectively. No. 6–9 set *T_on_*: 60 μs and SV: 45 V unchanged, and change *T_off_* to 26, 28, 32, and 34 μs, respectively. No. 10–13 set *T_on_*: 60 μs and *T_off_*: 30 μs unchanged, and change SV to 41, 43, 47, and 49 V, respectively.

**Figure 15:**
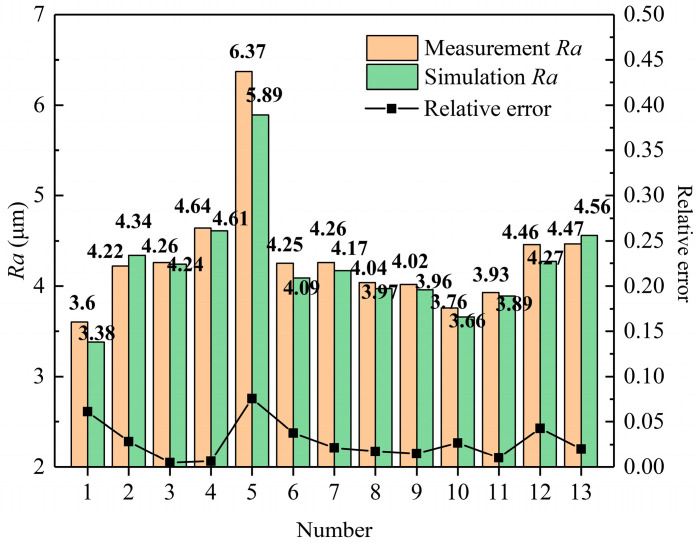
The comparison between the simulated and experimental data of *Ra*.

Figure 15 shows the comparative analysis between the simulated and experimental results of *Ra.* From Figure 15, the simulation values of *Ra* show good agreement with the experimental result, with a relative error of 0.47 to 7.54%. This indicates that the established thermophysical model during the MPDP has high accuracy, which can provide some guidance value for practical production, such as in the setting of discharge parameters.

(8) $$Ra=\frac{1}{n}\sum \left|y_{i}\right|$$

From Figure 15, *T_on_*, *T_off_*, and *SV* have a great influence on surface roughness. *Ra* increases with an increase in *T_on_* and *SV*, and with further increases in *SV*, *Ra* increases at a lower rate. This is mainly because the discharge energy of a single pulse increases with the increase in *T_on_* and *SV*. Continuous pulse discharge machining is the superposition effect of a large amount of SPDP machining. Higher pulse discharge energy will produce larger discharge craters and make the machined surface rougher. In addition, higher pulse discharge energy may promote the exposure and shedding of more SiC particles, thereby increasing the *Ra* on the machined surface. *Ra* slightly drops along with the increase in *T_off_*. This is mainly because an increase in pulse-off duration advantageously improves the flushing of the molten debris and molten metal accumulation in the inter-electrode gap and promotes the dielectric to take away more heat, exhibiting good surface quality.

## 7. Conclusions

1. The simulation results of the thermophysical model during the SPDP indicate that the shape of the melted or vaporized material is elliptical. The length in the radius direction is greater than the length in the depth direction. The highest temperature of the workplace exceeds the melting and boiling temperatures of the Al matrix and also exceeds the heat decomposition temperature of silicon carbide.
2. The thermophysical model simulation results and experimental characterization data during the MPDP indicate that the removal mechanism of SiCp/Al composite material consists of the melting and vaporization of aluminum matrix, as well as the heat decomposition and shedding of SiC particles.
3. The simulation values of *Ra* show good agreement with the experiment results, with a relative error of 0.47% to 7.54%. Namely, the established thermophysical model during the MPDP can provide some guidance value for practical production, such as in the setting of discharge parameters.

## Figures and Tables

**Table 1:** The main technical parameters of the machining equipment.

Parameters	Value or Content
Gap peak voltage	50–140 V
Maximum current value	100 A
Ton	0.1–100 μs
Toff	1–120 μs
Feed rate	0.1–500 mm^2^/min
Wire diameter	0.15–0.3 mm
WT	3–22 N
Dielectric	Deionized water

**Table 2:** The main parameters of the measuring equipment.

Contents	Equipment Type	Parameters
Surface roughness morphology (*Ra*)	WYKO NT9100	Longitudinal scanning range: 0.1 nm–1 mm; Longitudinal resolution: 0.1 nm
The microstructure and elemental distribution	MIRA 3 LMU	Magnification: 1000×; Resolution: 144 eV
The phase composition	Bruce D8 ADVANCE	Radiation range: 20–80°; Scanning step size: 0.045°/step

**Table 3:** The physical parameters of Al [35].

Thermal Properties	Value
Density (kg/m^3^)	2700
Specific heat (solid) (J/(kg·K))	900
Thermal conductivity (solid) (W/(m·K))	238
Melting point temperature (K)	933
Latent heat of melting (kJ/kg)	390
Evaporation point temperature (K)	2743
Latent heat of evaporation (kJ/kg)	11,834
Specific heat (liquid) (J/(kg·K))	1127
Thermal conductivity (liquid < 1491 K) (W/(m·K))	33.9 + 0.07892T − 2.099 × 10^−5^T^2^
Thermal conductivity (liquid > 1491 K) (W/(m·K))	105

**Table 4:** The physical parameters of SiC [35].

Thermal Properties	Value
Density (kg/m^3^)	3240
Decomposition temperature (K)	3100
Specific heat (solid) (J/(kg·K))	0.48 + 0.023exp(T/262)
Thermal conductivity (solid) (W/(m·K))	2.67 × 10^5^T^−1.26^
Latent heat of evaporation (kJ/kg)	13,250

**Table 5:** The chemical composition on the workpiece surface.

Region		Element				
		C	O	Al	Si	Cu
A	weight%	33.84 ± 1.69	2.72 ± 0.39	0.32 ± 0.04	63.03 ± 2.82	0.10 ± 0.02
	atomic%	53.72 ± 2.68	3.24 ± 0.32	0.22 ± 0.03	42.79 ± 2.13	0.03 ± 0.01
B	weight%	17.93 ± 1.78	14.76 ± 1.47	41.64 ± 2.08	24.68 ± 1.23	1.00 ± 0.15
	atomic%	30.76 ± 1.53	19.01 ± 1.88	31.8 ± 1.58	18.11 ± 1.79	0.32 ± 0.05

## Data Availability

The original contributions presented in the study are included in the article, further inquiries can be directed to the corresponding author.

## Nomenclature

**No.**	**Symbol**	**Definition**	**Unit**
1	*A*	Thermal conduction area	m^2^
2	Al	Aluminum	
3	CNC	Computer numerical control	
4	*dT*/*dδ*	Temperature gradient	K/m
5	EDM	Electrical discharge machining	
6	EDS	Energy dispersive spectrometer	
7	*f_c_*	Energy absorption coefficient of the workpiece	
8	*h*	Coefficient of thermal convection	W/(m^2^·K)
9	*I_(t)_*	Discharge current	A
10	MPDP	Multi-pulse discharge process	
11	*MRR*	Material removal rate	mm^2^/s
12	*q*	Thermal conduction per unit time	Q/s
13	*R*	Discharge radius	m
14	*Ra*	Surface roughness	μm
15	SEM	Scanning electron microscope	
16	SiC	Silicon carbide	
17	SiCp/Al	Silicon carbide particle reinforced aluminum matrix composite	
18	SPDP	Single-pulse discharge process	
19	*SV*	Servo voltage	V
20	*T_0_*	Room temperature	K
21	*T_off_*	Pulse-off time	μs
22	*T_on_*	Pulse-on time	μs
23	*U_(t)_*	Discharge voltage	V
24	WEDM	Wire electrical discharge machining	
25	*WS*	Wire speed	m/s
26	*WT*	Wire tension	N
27	XRD	X-ray diffraction	
28	*λ*	Thermal conductivity	W/(m·K)
